# A case of metastatic malignant granular cell tumour of the scalp

**DOI:** 10.1093/jscr/rjab145

**Published:** 2021-04-24

**Authors:** Geoffrey Williams, Carlos Neblett, Shereika Warren, Garfield Blake

**Affiliations:** 1 Department of Surgery, Division of Plastic and Reconstructive Surgery, Cornwall Regional Hospital, Montego Bay, Jamaica; 2 Department of Pathology, Cornwall Regional Hospital, Montego Bay, Jamaica

## Abstract

Granular cell tumours of the scalp are rare. Malignant transformation of these tumours is even more uncommon, making the diagnosis exceedingly difficult. The recommended treatment of surgical excision with negative margins is not easily achieved in this location, given the anatomy of the scalp.

## INTRODUCTION

Granular cell tumours (GCTs) are a rare form of neural derived neoplasm usually arising from Schwann cells [1]. These tumours most commonly occur in the head and neck region with a strong predilection for the tongue, but may occur in any part of the body [2, 3]. The vast majority (98%) of these tumours are benign, but even when malignant, are usually slow-growing and relatively small lesions [4]. The authors present a rare case of a malignant GCT of the scalp, representing the only such published case that we could find in a review of the literature.

## CASE PRESENTATION

A 60-year-old man presented to a hospital with swelling on his occipital scalp in 2012. He underwent wide local excision of the mass, including the pericranium, with immediate reconstruction using a local scalp flap and a split-thickness skin graft to the donor area of the flap.

Histopathological examination of the mass demonstrated features consistent with a malignant GCT: surface ulceration, dermal nests and sheets of round, polygonal and spindled cells with abundant granular eosinophilic cytoplasm with nuclear hyperchromasia and pleomorphism. Importantly, the tumour was noted to have invaded the pericranium.

**Figure 1:**
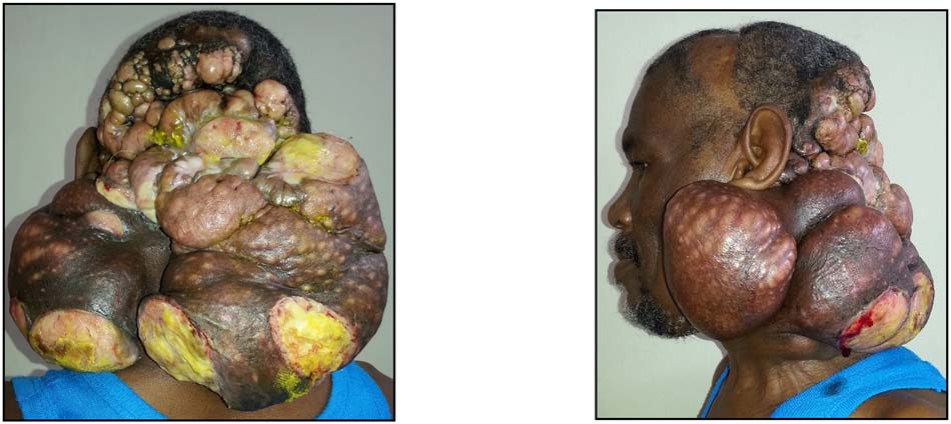
Posterior and left lateral clinical photographs of the recurrent malignant GCT.

**Figure 2:**
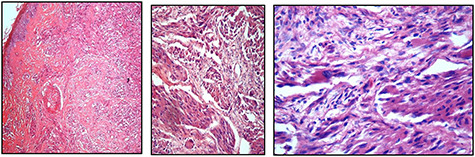
**(A**, **B**, and **C**) Showing the evidence of cellular spindling, hyperchromasia and nuclear pleomorphism, features in keeping with malignant GCT, ranging from low to high magnifications, respectively

The patient was lost to follow-up and re-presented 3 years later with a massive fungating scalp mass (Fig. 1). An incisional biopsy done at this time confirmed the clinical diagnosis of a recurrence of the tumour (Fig. 2).

In addition to the obvious large fungating mass of the scalp, physical examination also revealed multiple cervical nodes in zones II-V on the right side; the left side was not amenable to palpation due to the presence of the mass (Fig. 1).

A staging magnetic resonance imaging study showed a heterogenous soft tissue mass in the occipito-cervical region measuring 16 × 21 cm, with no evidence of intracranial extension. A plain chest radiograph showed multiple lesions of variable sizes in both lung fields indicative of metastatic disease. An abdominal ultrasound revealed a targetoid 2.3 × 2.0 x 1.9 cm hypoechoic lesion in segment VII of the liver, suspicious for metastasis.

An assessment of unresectable, terminal metastatic malignant GCT of the scalp was made and the patient recommended for palliative care.

## DISCUSSION

GCTs are localised mainly in the dermis, subcutis or submucosa of various locations—respiratory, digestive or urinary tract. They are predominantly benign, with only 1–2% undergoing malignant transformation [5–7].

Histological features of malignant change, as proposed by Fanburg-Smith *et al*. include the following six findings—(i) necrosis, (ii) cellular spindling, (iii) vesicular nuclei with large nuclei, (iv) increased mitotic activity, (v) high nuclear to cytoplasmic ratio and (vi) pleomorphism [5, 6]. A minimum of three of the six features are required for a lesion to be diagnosed as malignant, whereas those with less than three are termed as atypical [5, 6]. Immunohistochemical analysis has shown consistent positivity for S-100 protein that supports the view that GCT is of peripheral nerve sheath origin [8–10].

The question as to whether GCTs arises *de novo* or from the transformation of benign lesions is still unsettled. Chen *et al*. [11] challenged the prevailing dogma that malignant GCTs arises *de novo* and postulated that benign lesions can undergo malignant transformation.

Clinically, it has been noted by some authors that malignant GCTs behave in a very aggressive manner, similar to high-grade sarcomas [5–7].

Malignant GCTs typically grow rapidly, often ulcerate, invade locally and tend to spread with extensive metastases; mainly to the lymph nodes, lungs and bones and rarely to the intestines, liver and brain. The aim of treatment for malignant GCT is complete surgical excision, where possible. There is no established role for chemotherapy or radiotherapy [12].

In the index case with the extent of the scalp mass and metastases to the lymph nodes, lungs and liver, the treatment option was only one of palliation. After review by a multidisciplinary tumour board, we accepted the view of the oncologist that we attempt palliation as is done for sarcomas, which these tumours mimic in behaviour. The patient was therefore started on a course of pazopanib, a potent and selective multi-targeted receptor tyrosine kinase inhibitor that blocks tumour growth and is approved for soft tissue sarcoma treatment. However, there was no response to the drug and the patient soon demised.

Malignant transformation of GCTs is exceedingly rare with less than a hundred cases reported in the literature. In addition, the scalp is a rare anatomical site for GCTs in general with only one other case reported in the literature which was benign [13]. This report represents the only case of a malignant GCT of the scalp.

## CONFLICT OF INTEREST STATEMENT

None declared.

## FUNDING

None.
